# Effect of Emotion on Prospective Memory in Those of Different Age Groups

**DOI:** 10.1155/2020/8859231

**Published:** 2020-09-21

**Authors:** Jinhua Xian, Yan Wang, Buxin Han

**Affiliations:** ^1^Center on Aging Psychology, CAS Key Laboratory of Mental Health, Institute of Psychology, Beijing, China; ^2^School of Education Science, Jiangsu Second Normal University, Nanjing, China; ^3^Department of Psychology, University of Chinese Academy of Sciences, Beijing, China

## Abstract

The effect of emotion on prospective memory on those of different age groups and its neural mechanism in Chinese adults are still unclear. The present study investigated the effect of emotion on prospective memory during the encoding and retrieval phases in younger and older adults by using event-related potentials (ERPs). In the behavioral results, a shorter response time was found for positive prospective memory cues only in older group. In the ERP results, during the encoding phase, an increased late positive potential (LPP) was found for negative prospective memory cues in younger adults, while the amplitude of the LPP was marginally greater for positive prospective memory cues than for negative prospective memory cues in older adults. Correspondingly, younger adults showed an increased parietal positivity for negative prospective memory cues, while an elevated parietal positivity for positive prospective memory cues was found in older adults during the retrieval phase. This finding reflects the increased attentional processing of encoding and the more cognitive resources recruited to carry out a set of processes that are associated with the realization of delayed intentions when the prospective memory cues are emotional. The results reveal the effect of emotion on prospective memory during the encoding and retrieval phases in Chinese adults, modulated by aging, as shown by a positivity effect on older adults and a negativity bias in younger adults.

## 1. Introduction

Prospective memory (PM) refers to remembering to execute delayed intentions in the future [1, 2]. Difficulties in prospective memory adversely impact the quality of life of older adults who experience problems independently managing their instrumental activities of daily living, for example, forgetting to take medications [3].

In laboratory-based prospective memory tasks, participants need to perform an intended action after the recognition of an external cue in the environment [4]. Regarding the age-related decrease in executive functions and episodic memory [5], some studies have revealed a decline in prospective memory with increasing age [6–8]. However, when more salient cues are presented to participants, older adults perform better prospective memory [9, 10]. Additionally, this age-related decline has not been found in naturalistic PM tasks, which has been labeled the “age-PM paradox” [11]. Regarding the reason for this paradox, the stimuli in naturalistic tasks usually seem to be emotional and may contain personal or social meanings. Therefore, emotional PM cues may enhance prospective memory performance in older adults through their emotional valence.

Considering the ecological validity of studies, researchers have been interested in the influence of emotional PM cues on prospective memory in younger and older adults, obtaining inconsistent results. Some studies have shown better prospective memory performance for positive and negative PM cues in both age groups [12]. However, other studies have shown that positive and negative PM cues improve prospective memory performance only for older adults [4, 13]. Moreover, in still other research, compared with neutral PM cues, better prospective memory performance was found only for positive PM cues and not for negative PM cues in both younger and older adults [14]. Therefore, the effects of emotion on prospective memory among those of different age groups are still unclear.

Similarly, a growing body of the literature has revealed that emotion affects attention and retrospective memory [15–17]. Furthermore, different effects of emotion have been found in younger and older adults. According to socioemotional selectivity theory, older adults tend to remember more positive stimuli than negative stimuli, which is called the positivity effect. Because the future is limited for older adults, positive stimuli are considered more emotionally meaningful [18]. However, a negativity preference has been found in younger adults [19, 20]. Such effects of emotion on retrospective memory can be found not only in Western adults but also in Eastern adults (Korea and China) [21, 22]. Another research has compared visual attention to emotional and neutral facial expressions in Chinese adults by using eye-tracking techniques. The results revealed no attentional preferences for positive stimuli in older adults [23]. Therefore, the emotional enhancement effect between Western and Eastern adults is inconsistent. According to our current search of the literature, studies on the effects of emotion on prospective memory were all conducted in Western adults [4, 12, 13]. The effect of emotion on prospective memory in Chinese adults needs to be clarified.

To explain the mechanism of age-related effects of emotion on prospective memory, a few studies have used event-related potentials (ERPs) to demonstrate the different effects of emotion on prospective memory in younger and older adults. For example, one study examined the influence of emotional PM cues on prospective memory only in younger adults [24]. Another study further examined it in younger and older adults [25]. During encoding, the late positive potential (LPP), which is located in the central and parietal regions, begins approximately 300 ms after stimulus onset and lasts for 1000–2000 ms. It has been shown to reflect attentional processes [26]. Hering et al. [25] found an elevated LPP for emotional PM cues for both age groups, indicating an increased attentional processing of encoding emotional PM cues. Moreover, the elevated activity for unpleasant PM cues was found in younger adults. During retrieval, the N300, which appears between 300 and 500 ms after the onset of a prospective memory cue, is located in the occipital-parietal region. It is associated with the detection of PM cues [27]. Parietal positivity, which appears between 400 and 1200 ms after the onset of a prospective memory cue, is located in the parietal region. It reflects three components related to the detection of low probability targets (P3b), the recognition of prospective memory cues (parietal old-new effect), and the configuration of the prospective memory task set (prospective positivity). Parietal positivity represents a set of processes that are associated with the realization of delayed intentions [27]. Some studies have revealed that emotional materials modulate parietal positivity [24, 25]. Furthermore, Hering et al. [25] found increased activity of ERP components for pleasant PM cues in older adults.

The aim of this study is to investigate the effect of emotion on prospective memory among those of different age groups and its neural mechanism in Chinese adults. Following previous studies [22, 25], we expect that, given their general deficits in cognition, older adults' prospective memory performance will benefit from emotional PM cues. Moreover, we expect younger adults to have better prospective memory performance than older adults. Regarding the ERP results, we expect to find an effect of emotion on prospective memory during the encoding and retrieval phases, with a positivity effect on older adults and a negativity bias in younger adults. We expect elevated ERP activities (LPP and parietal positivity) for positive PM cues relative to negative and neutral PM cues in older adults, while we expect increased amplitudes of the LPP and parietal positivity for negative PM cues relative to positive and neutral PM cues in younger adults.

## 2. Methods

### 2.1. Participants

The experiment was conducted in accordance with the Declaration of Helsinki and was approved by the Ethics Committee of the Institute of Psychology, Chinese Academy of Sciences. All participants signed an informed consent form and received monetary compensation for their participation.

A total of 24 young adults from local universities (age *M* = 20.9, SD = 1.9; 18–24 years; 13 female) and 23 community-dwelling older adults were included in the experiment (age *M* = 72.2, SD = 5.4; 65–83 years; 10 female). All participants had normal or corrected-to-normal vision and no neurological or psychiatric pathologies. None of the participants reported having phobic fears (e.g., ophidiophobia) related to our study materials. Moreover, the Mini-Mental State Examination (MMSE) test for general cognitive status was used for all older adults. Only those with scores above 27 were allowed to participate in our test (*M* = 28.61, SD = 1.03) [28, 29].

The two groups were matched by the educational level, *t*(45) = 0.07, *p* > 0.05; self-rated general health, *t*(45) = 1.74, *p* > 0.05; self-rated mental health, *t*(45) = 0.53, *p* > 0.05; and initial mood (as measured before the experiment), *t*(45) = 0.44, *p* > 0.05 (Table 1). Their health, mental health, and initial mood were all measured on a 5-point rating scale, with 1 as very bad and 5 as very good.

### 2.2. Materials

This study used the paradigm of Cona et al. [24], the prospective memory task comprised an ongoing task with a prospective memory instruction, to remember to make a key-press when specific stimulus occurred [25]. All 213 pictures were selected from the International Affective Pictures System [30, 31]. The 1-back visual working memory paradigm was used as the ongoing task which included 51 positive (*M* = 6.58, SD = 1.29), 51 negative (*M* = 3.63, SD = 1.31), and 51 neutral pictures (*M* = 5.00, SD = 1.34). There were 20 positive PM cues (*M* = 6.91, SD = 1.33), 20 negative PM cues (*M* = 3.08, SD = 1.26), and 20 neutral PM cues (*M* = 5.01, SD = 1.32). The emotional arousal levels of pictures were kept at the middle of the arousal scale (ongoing task stimuli: *M* = 4.73, SD = 1.72; prospective memory cues: *M* = 4.73, SD = 1.83) [4]. The emotional valence and arousal ratings were taken from the ratings that were natively assessed by Huang and Luo [31]. Each picture (4.7° × 6.3°) was displayed in the center of a black screen.

### 2.3. Procedure

The whole experiment included three sessions, which varied based on the emotional valence of the PM cue (positive, negative, and neutral) [24]. Each session contained 5 blocks, and each block contained 73 ongoing stimuli and 8 PM cues (four PM cues were repeated) for a total of 405 stimuli with equal proportions of positive, negative, and neutral pictures. In each trial, a fixation cross randomly lasted 1200, 1400, or 1600 ms [24, 25], followed by the stimulus, which was displayed for a maximum of 3000 ms or until a response was made. All stimuli were presented in a fixed pseudorandomized order. In the course of the ongoing task, in each session, 96 one-back hits (24% of 405 stimuli) were presented. The PM cues were never one-back hits. The order of the three sessions was counterbalanced across participants [4, 24].

During the test, the participants were asked to carry out the 1-back visual working memory task, which involved deciding whether the picture occurring on the screen was the same (by pressing the “N” key) or different (by pressing the “M” key) from the picture occurring one trial before. After finishing 39 1-back visual working memory practice trials, the participants were informed that later in the session, they needed to complete two equally important tasks simultaneously. Then, the 1-back visual working memory instructions and prospective memory instructions were shown. The participants needed to remember to press the “V” key whenever predefined PM cues were displayed. They were asked to explain the task in their own words before they studied these PM cues. Before each block, four PM cues were displayed randomly one by one on the screen. When one session was finished, the participants were asked to identify the 20 PM cues among 20 distractors as a recognition task. E-Prime (V2; Psychology Software Tools, Inc., Pittsburgh, PA) was used to implement the experiment.

### 2.4. Electrophysiological Recording

EEG data were recorded from 64 Ag/AgCl electrodes mounted in an elastic cap (Neuroscan Inc.). The physical reference electrode was approximately 2 cm posterior to the CZ, and the EEG data were rereferenced to the average of the left and right mastoids. Vertical and horizontal eye movements were recorded from electrodes placed below and beside the eyes. Electrode impedance was kept below 5 kΩ. Data were digitized at a sampling rate of 500 Hz, applying a filter bandwidth of 0.05–100 Hz.

### 2.5. Data Analysis

Data were analyzed with SPSS (SPSS, Inc., Chicago) for Windows. *p* < 0.05 was considered statistically significant. The prospective memory accuracy and response time for correct PM cue responses were analyzed by a mixed 2 × 3 ANOVA, with the age group (younger and older) as the between-subject factor and emotional valence of PM cues (positive, neutral, and negative) as the within-subject factor.

ERP epochs were extracted offline and included 200 ms of prestimulus activity and 1400 ms of poststimulus activity. The ERP data were digitally filtered with a 30 Hz low pass, and baseline correction was made using the prestimulus 200 ms interval. Epoch rejection was performed with a criterion of ±100 *μ*V. ERPs were averaged for correct PM cue trials.

The selection of epochs and electrodes for the analyses was guided by previous studies [24, 32, 33]. The amplitude of the LPP was measured as the mean activity between 400 and 650 ms and included data from six electrodes: PO3, PO4, PO5, PO6, PO7, and PO8. For the analyses, the electrodes within one hemisphere were collapsed to obtain a mean activity. The amplitude of the N300 was measured as the mean activity between 270 and 350 ms and included six electrodes: PO3, PO4, PO5, PO6, PO7, and PO8. The amplitude of the parietal positivity was measured as the mean activity between 500 and 800 ms and included six electrodes: P1, P2, P3, P4, P5, and P6. The ANOVA factors included age group (younger and older), emotional valence of PM cues (positive, neutral, and negative) and hemisphere (left and right). Greenhouse–Geisser correction was used to compensate for sphericity violations. Simple effect analyses were conducted to explore the interaction effects.

## 3. Results

### 3.1. Behavioral Results

The prospective memory performance for younger and older adults is presented in Table 2. The analysis of prospective memory accuracy revealed significant main effects of emotional valence of PM cues and age group [*F*(2, 90) = 4.89, *p* < 0.05, *η*^2^ = 0.10; *F*(1, 45) = 13.79, *p* < 0.01, *η*^2^ = 0.23]. The interaction between age group and emotional valence of PM cues was significant [*F*(2, 90) = 3.93, *p* < 0.05, *η*^2^ = 0.08], revealing higher accuracy for both positive and negative PM cues relative to neutral PM cues for older adults (*ps* < 0.01), but not for younger adults. The accuracy in prospective memory performance was lower for older adults than for younger adults in all conditions [*F*(1, 45) = 6.49, *p* < 0.05, *η*^2^ = 0.13; *F*(1, 45) = 12.81, *p* < 0.01, *η*^2^ = 0.22; *F*(1, 45) = 4.52, *p* < 0.05, *η*^2^ = 0.09].

The analysis of prospective memory response time revealed the significant main effects of emotional valence of PM cues and age group [*F*(2, 90) = 3.38, *p* < 0.05, *η*^2^ = 0.07; *F*(1, 45) = 36.47, *p* < 0.001, *η*^2^ = 0.45]. The interaction between age group and emotional valence of PM cues was significant [*F*(2, 90) = 6.04, *p* < 0.01, *η*^2^ = 0.12], revealing shorter response times for positive PM cues than for negative and neutral PM cues for older adults (*ps* < 0.05), but not for younger adults. The response times in prospective memory were longer for older adults than for younger adults in the three conditions [*F*(1, 45) = 16.19, *p* < 0.001, *η*^2^ = 0.27; *F*(1, 45) = 43.85, *p* < 0.001, *η*^2^ = 0.49; *F*(1, 45) = 31.70, *p* < 0.001, *η*^2^ = 0.41].

### 3.2. ERP Results

#### 3.2.1. Encoding Phase

**Figure 1:**
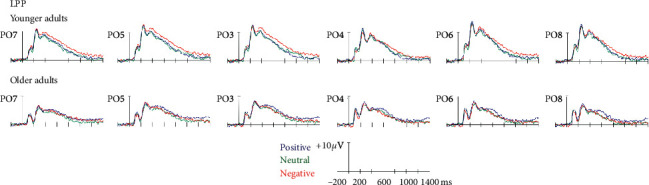
Encoding phase. Grand average event-related potentials at 6 electrodes used in the ANOVA, demonstrating the LPP (400–650 ms, PO3, PO4, PO5, PO6, PO7, and PO8) elicited by positive, neutral, and negative PM cues in younger and older adults.

*LPP*: the main effect of age group was significant [*F*(1, 45) = 6.80, *p* < 0.05, *η*^2^ = 0.13], reflecting the increased amplitude for younger adults compared to older adults. The interaction between emotional valence of PM cues and age group was significant [*F*(2, 90) = 4.44, *p* < 0.05, *η*^2^ = 0.09], reflecting the greater amplitude for negative PM cues than for neutral and positive PM cues in younger adults (*ps* < 0.05), and the marginally greater amplitude for positive PM cues than for negative PM cues in older adults (*p* = 0.067). The interaction between emotional valence of PM cues and hemisphere was significant [*F*(2, 90) = 4.48, *p* < 0.05, *η*^2^ = 0.09], reflecting a greater amplitude for negative PM cues than for neutral PM cues in the left hemisphere (*p* < 0.05) (Figure 1, Table 3).

#### 3.2.2. Retrieval Phase

**Figure 2:**
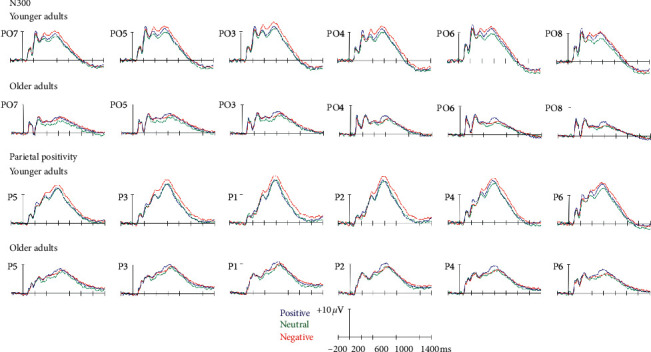
Retrieval phase. Grand average event-related potentials at 12 electrodes used in the ANOVA, demonstrating the N300 (270–350 ms, PO3, PO4, PO5, PO6, PO7, PO8) and parietal positivity (500–800 ms, P1, P2, P3, P4, P5, P6) elicited by positive, neutral, and negative PM cues in younger and older adults.

*N300*: the main effect of emotional valence of PM cues was significant [*F*(2, 90) = 7.27, *p* < 0.01, *η*^2^ = 0.14], reflecting the attenuated amplitude for positive and negative PM cues compared to neutral PM cues (*ps* < 0.05). The main effect of age group was significant [*F*(1, 45) = 10.28, *p* < 0.01, *η*^2^ = 0.19], reflecting more expressed amplitude in older adults than in younger adults. The interaction between emotional valence of PM cues and hemisphere was significant [*F*(2, 90) = 5.27, *p* < 0.01, *η*^2^ = 0.11], reflecting more expressed amplitude for neutral PM cues than for positive and negative PM cues in the left hemisphere (*ps* < 0.01), and more expressed amplitude for neutral PM cues than for positive PM cues in the right hemisphere (*p* < 0.01) (Figure 2, Table 3).

Parietal Positivity: the main effect of emotional valence of PM cues was significant [*F*(2, 90) = 9.28, *p* < 0.001, *η*^2^ = 0.17], reflecting an elevated parietal positivity for negative PM cues than for neutral PM cues (*p* < 0.001). The main effect of age group was significant [*F*(1, 45) = 12.63, *p* < 0.01, *η*^2^ = 0.22], reflecting an elevated parietal positivity for younger adults than for older adults. The interaction between emotional valence of PM cues and age group was significant [*F*(2, 90) = 10.32, *p* < 0.001, *η*^2^ = 0.19], reflecting an elevated parietal positivity for negative PM cues compared to neutral and positive PM cues in younger adults (*ps* < 0.001) and an elevated parietal positivity for positive PM cues compared to neutral PM cues in older adults (*p* < 0.05). The interaction between age group and hemisphere was significant [*F*(1, 45)= 5.15, *p* < 0.05, η^2^= 0.10], reflecting an elevated parietal positivity in the left hemisphere than in the right hemisphere in older adults (*p* < 0.05). The interaction between emotional valence of PM cues and hemisphere was significant [*F*(2,90) = 4.15, *p* < 0.05, *η*^2^ = 0.08], reflecting an elevated parietal positivity for negative PM cues compared to neutral and positive PM cues in the left hemisphere (*ps* < 0.05), and an elevated parietal positivity for negative and positive PM cues compared to neutral PM cues in the right hemisphere (*ps* < 0.05) (Figure 2, Table 3).

## 4. Discussion

The aim of our study was to investigate the effect of emotion on prospective memory in younger and older Chinese adults by using ERPs. Higher accuracy of prospective memory was found for positive/negative PM cues than for neutral PM cues in older adults, which was in line with previous studies showing that emotional PM cues could improve older adults' prospective memory performance through high salience [4, 13]. However, the accuracy of prospective memory in younger adults was generally high, and no effect of emotion was found, which is consistent with previous studies [4, 13, 24]. This may be because the task we used was easy. Due to the ERP approach of our study, we decided to use the easy prospective memory task to avoid having to reject too many epochs, especially for older adults [25]. Furthermore, the response times of prospective memory were significantly different only for older adults, with shorter response times for positive PM cues than for negative and neutral PM cues. This finding seems to indicate a positivity effect on prospective memory in older adults [18].

The ERP data also showed this effect of emotion on prospective memory in younger and older adults during the encoding and retrieval phases. As hypothesized, the emotional manipulation started from the encoding phase of prospective memory. The interaction effect between aging and emotion was found, as evidenced by the increased LPP for negative PM cues compared to neutral and positive PM cues in younger adults, while the amplitude of the LPP was marginally greater for positive PM cues than for negative PM cues in older adults. Moreover, the results revealed a greater LPP for negative PM cues over left occipital-parietal regions. This finding may indicate that the emotional valence of PM cues affects the encoding of intention differently in the hemispheres. Given that the LPP is related to attention to emotional information [26, 34], our findings indicate that both age groups had increased attentional processing of encoding the emotional PM cues. Furthermore, the emotional materials differently affected younger and older adults' prospective memory, with a negativity bias in younger adults and a positivity effect on older adults.

Regarding the retrieval phase, attenuated amplitudes of the N300 were found for emotional PM cues compared to neutral PM cues in both age groups. As mentioned before, the N300 is associated with the detection of PM cues [27]. This finding reflects that emotional materials affected cue detection in both age groups. Moreover, consistent with a previous study [25], N300 was more expressed in older adults than in younger adults. However, another study revealed attenuated activity in older adults compared to younger adults [35]. Others did not find any age-related differences in the amplitude of the N300 [32, 36]. This may be because the stimuli in our study were emotional.

In addition, an interaction effect between aging and emotion on prospective memory was also found during the retrieval phase, as evidenced by an elevated parietal positivity for negative PM cues compared to neutral and positive PM cues in younger adults, while there was an increased parietal positivity for positive PM cues compared to neutral PM cues in older adults. The results also revealed an elevated parietal positivity for negative PM cues over left parietal regions, and an elevated parietal positivity for negative and positive PM cues over right parietal regions. This finding seems to indicate the effect of emotion on prospective memory across hemispheres during the retrieval phase. As mentioned before, parietal positivity reflects three components related to the detection of low probability targets (P3b), the recognition of prospective memory cues (parietal old-new effect), and the configuration of the prospective memory task set (prospective positivity) [27]. Our results demonstrated an increased allocation of resources to carry out a set of processes that are associated with the realization of delayed intentions when the PM cues were emotional [24]. This may be because emotional information receives increased processing due to motivational factors [26, 37].

Taken together, our study revealed an effect of emotion on prospective memory in Chinese adults, showing a positivity effect on older adults and a negativity bias in younger adults. Furthermore, this effect of emotion on prospective memory started from the encoding phase, increased the attentional processing of encoding emotional PM cues and then recruited more resources to accomplish the realization of delayed intentions during the retrieval phase. These findings in Chinese adults are similar to those of a study based on Western participants [25], showing the similar effect of emotion on the encoding and retrieval phases of prospective memory in Chinese adults. According to socioemotional selectivity theory [18, 20], our results suggest that older adults serve the positivity effect as an emotion regulatory strategy. Following previous studies [19, 38], a preference for negativity was found in younger adults, which may be because they are prone to show the stronger evolutionary demands of negative (e.g., death) stimuli [39]. Moreover, our findings were inconsistent with those of Fung et al. [23], who found no attentional preferences for positive stimuli in older Chinese adults by comparing visual attention to emotional and neutral facial expressions. This maybe because performing a prospective memory task requires several cognitive processes, not just attention. Therefore, an emotional enhancement effect on prospective memory could be found in Western and Eastern adults.

As stated before, the prospective memory task is critical for independent living, which is a particularly important issue for older adults. Our study investigated the effect of emotion on prospective memory in different age groups by using ERPs, showing older adults' prospective memory performance could be enhanced by manipulating the emotional valence of PM cues. Moreover, the emotional valence of PM cues affects prospective memory during the encoding and retrieval phases. These results could be useful to improve the quality of life by carrying out prospective memory training in older adults. Recently, brain computer interface is being one of the most popular technologies to improve the quality of life among older adults by enhancing or repairing their cognitive function and motor function [40]. Some studies have revealed the brain computer interface training system could improve memory and attention in older adults [41, 42]. Therefore, future research could use a brain computer interface application to train older adults to add personally positive emotions to PM cues when performing the prospective memory task, and the intervention should start from the encoding phase of prospective memory.

In terms of possible limitations, compared with ongoing task stimuli, the number of PM cues is relatively small, and the results need further verification. This is, however, limited by the characteristics of prospective memory and the more experimental conditions. Second, because the overall potential of the N300 in older adults was quite small, the age difference in the N300 also needs further verification. Future studies should use neuroimaging methods to discuss spatial sources for the effect of emotion on prospective memory in younger and older adults.

## 5. Conclusions

The present results suggest that the mechanism of prospective memory is affected by emotional materials in Chinese adults, as evidenced by an increased LPP and parietal positivity for emotional PM cues. This finding reflects the increased attentional processing of encoding and the more cognitive resources recruited to carry out a set of processes that are associated with the realization of delayed intentions when the PM cues are emotional. Moreover, this effect of emotion on prospective memory during the encoding and retrieval phases in Chinese adults is modulated by aging, as shown by a positivity effect on older adults and a negativity bias in younger adults. These findings will be helpful in enhancing older adults' quality of life by improving their prospective memory performance.

## Figures and Tables

**Table 1:** Demographic data for younger and older adults.

Demographic data	Younger adults (*n* = 24)		Older adults (*n* = 23)	
	*M*	SD	*M*	SD
Age	20.9	1.9	72.2	5.4
Years of education	14.0	1.5	14.0	2.5
Self-rated health	4.1	0.6	3.8	0.6
Self-rated mental health	4.0	0.6	4.0	0.6
Initial mood	3.4	0.6	3.4	0.5

**Table 2:** Prospective memory performance for younger and older adults.

Age group	Emotional valence of PM cues		
	Positive	Neutral	Negative
*Accuracy (SD)*			
Younger adults (*n* = 24)	0.98 (0.04)	0.97 (0.06)	0.97 (0.05)
Older adults (*n* = 23)	0.94 (0.07)	0.88 (0.10)	0.94 (0.05)
*Response time (SD)*			
Younger adults (*n* = 24)	896.83 (111.41)	886.57 (108.02)	882.62 (99.49)
Older adults (*n* = 23)	1057.18 (158.64)	1123.66 (136.36)	1157.04 (216.14)

**Table 3:** Grand average of LPP, N300, and parietal positivity.

Age group	Emotional valence of PM cues					
	Positive (L)	Neutral (L)	Negative (L)	Positive (R)	Neutral (R)	Negative (R)
*LPP (μV)*						
Younger adults (*n* = 24)	7.51 (4.06)	7.15 (3.35)	8.75 (3.83)	7.59 (5.29)	7.45 (4.27)	8.14 (5.84)
Older adults (*n* = 23)	5.84 (3.75)	4.92 (3.80)	5.15 (3.32)	5.28 (3.84)	4.33 (3.53)	4.33 (3.50)

*N300 (μV)*						
Younger adults (*n* = 24)	8.58 (4.20)	7.91 (4.29)	8.58 (4.61)	8.74 (4.70)	7.67 (5.11)	8.02 (5.07)
Older adults (*n* = 23)	5.15 (3.41)	4.41 (3.27)	5.35 (3.41)	4.70 (3.62)	4.38 (3.42)	4.61 (3.83)

*Parietal positivity (μV)*						
Younger adults (*n* = 24)	10.94 (4.25)	11.23 (4.54)	12.90 (3.98)	11.79 (5.09)	11.35 (5.45)	13.28 (5.16)
Older adults (*n* = 23)	8.81 (3.55)	7.73 (3.29)	8.37 (3.39)	8.14 (3.57)	6.97 (3.33)	7.26 (3.25)

## Data Availability

The data used to support the findings of this study are available from the corresponding author upon request.
